# Case report: Neuroendocrine breast carcinoma with a germline EGFR T790M mutation

**DOI:** 10.3389/fonc.2023.1176868

**Published:** 2023-05-17

**Authors:** Olivia A. Sagan, Anna Rothstein, Bhaghyasree Jambunathan, Mersiha Hadziahmetovic, Anita Antoniolli, M. Hammad Rashid

**Affiliations:** ^1^ The University of Toledo College of Medicine and Life Sciences, Toledo, OH, United States; ^2^ Promedica Physicians, Sylvania, OH, United States; ^3^ University of Toledo Medical Center – Dana Cancer Center, Toledo, OH, United States

**Keywords:** neuroendocrine breast cancer, germline EGFR T790M mutation, triple-negative breast cancer (TNBC), EGFR mutation, chemotherapy

## Abstract

**Background:**

The epidermal growth factor receptor (EGFR) p.Thr790Met (T790M) mutation was discovered as a resistance mechanism in patients with lung cancer treated with first- and second-generation tyrosine kinase inhibitors. Further studies revealed the EGFR T790M mutation in treatment-naive non-small cell lung carcinoma (NSCLC) and as a rare germline mutation strongly associated with NSCLC. Somatic EGFR T790M mutations have been reported in a limited population of patients with triple-negative breast cancer. There are no previous reports of a germline EGFR T790M mutation found in a patient with breast cancer.

**Case presentation:**

We present a rare case of a 42-year-old woman with a rapidly progressing 8 cm mass in the right lateral breast. An additional right breast mass with multiple lymph nodes characteristic or suspicious of metastasis was found. Ultrasound-guided biopsy showed high-grade, poorly differentiated invasive neuroendocrine carcinoma of the right breast and metastatic carcinoma of a right axillary lymph node. Genetic testing revealed a germline EGFR T790M mutation. The patient underwent neoadjuvant chemotherapy, right mastectomy with lymph node dissection, adjuvant radiation to the right chest wall and axilla, and adjuvant chemotherapy.

**Conclusion:**

This is the first reported case of a patient with high-grade neuroendocrine carcinoma, triple-negative breast cancer and a germline EGFR T790M mutation. Further investigation is needed to find a possible correlation between the cancer in this patient and her mutation. Since there are no current guidelines, further research is also needed to define screening protocols for patients with germline EGFR T790M mutations. Additional treatment options and cancer risk could also be found with further research, which would benefit all patients with a germline EGFR T790M mutation.

## Background

As our understanding of molecular genetics in health care expands, specific gene mutations have been characterized and targeted for various cancer treatments. One of these genes is the epidermal growth factor receptor (EGF-receptor or EGFR) gene. The EGF receptor actuates several downstream pathways that regulate a variety of different cellular processes, including DNA synthesis and cell proliferation, which makes it crucial for cancer development (1, 2). In 2004, the presence of activating somatic mutations in the EGF receptor tyrosine kinase domain was identified in non-small cell lung carcinoma (NSCLC) after seeing an increased response in patients treated with wild-type EGFR tyrosine kinase inhibitors (TKI) compared to standard platinum-based chemotherapy (3–7). These mutations are found on exon 19 (deletion), exon 20 (insertion), and exon 21 (L858R point mutation) (8–15). These developments led to first- and second-generation TKIs becoming the standard of care for patients with EGFR-mutated NSCLC (6, 7, 16–18).

However, a specific subset of patients with NSCLC was found to progress while on first- or second-generation TKI. In more than 50% of these patients, the point mutation of EGFR p.Thr790Met (T790M) on Exon 20 was acquired after exposure to TKI, making the first- and second-generation TKI ineffective (19–21). Osimertinib was approved in 2018 as a third-generation TKI for any patient with EGFR-mutated NSCLC who had progressed to first- or second-generation TKI, regardless of the EGFR T790M mutation status (22). Although most patients develop the EGFR T790M mutation after treatment with TKIs, there have been documented cases of patients with treatment-naive EGFR T790M positive NSCLC. The prevalence of the EGFR T790M mutation has been reported between less than 5% and 40% in treatment-naive NSCLC (11, 23–27). The prevalence can vary greatly depending on the study and the technique used (28). Even with this variation, it is clear that the EGFR T790M mutation is not simply a post-TKI treatment resistance mechanism. Although these reports are rare, cases of germline EGFR T790M mutations have also been documented (29, 30). These cases have been of isolated individuals and families, and up to 90% of these patients have been diagnosed with lung adenocarcinoma (30–39).

The EGFR gene also plays an important role in breast cancer (BC). When the EGFR gene is abnormally expressed in BC, it is usually overexpressed or amplified. Although there have been isolated cases of activation of EGFR mutations in BC, this is very rare (3, 40–50). To investigate the role of TKIs in BC, the prevalence of EGFR T790M mutations has been researched. Few patients were found to have this somatic mutation, mainly in patients with triple-negative BC (TNBC) (50, 51). There have not been previously documented cases of patients with a germline EGFR T790M mutation and BC. The importance of the EGFR gene in BC has been clearly defined, but the extent and prevalence of the EGFR T790M mutation in BC have not yet been fully determined.

We present a rare and interesting case of a young woman who developed high-grade neuroendocrine carcinoma, TNBC and was found to have a germline EGFR T790M mutation.

## Case presentation

Our patient is a 42-year-old woman who initially presented with a small palpable mass on her right breast with accompanying burning pain. She stated that it had been growing rapidly over 6 to 8 weeks. She also reported night sweats, back pain, headaches, depression, anxiety, and memory loss. In particular, her respiratory review of the systems was negative and she had no smoking history. The patient has a history of hypertension, depression, intramural uterine leiomyoma, and a left breast fibroadenoma removed 20 years ago. In particular, her family history of cancer includes her daughter (leukemia) and her maternal aunt (colon cancer). Her father’s medical history is unknown. An initial physical exam showed swelling of the right breast. Palpation demonstrated an 8 cm mass in the right upper outer and lower outer quadrants and a walnut-sized mass in the right axillary tail. No tenderness, skin d’orange appearance, nipple discharge, or supraclavicular lymphadenopathy were documented.

The further imaging was promptly completed. A diagnostic mammogram with ultrasound of the right breast showed an irregular hypoechoic mass of 2.9 x 2.1 x 1.9 cm in the position of 10:00 of the right breast, which was highly suggestive of malignancy. An ultrasound of the right axilla showed an abnormally enlarged lymph node (LN) measuring 2.8 x 1.9 x 1.5 cm with an abnormal cortical thickness of 1 cm. This was noted to be highly concerning for a metastatic LN. An ultrasound-guided biopsy of the right breast mass and the right axillary LN was performed. The specimen of the right breast mass showed invasive high-grade neuroendocrine carcinoma of the breast with extensive necrosis. The LN was positive for metastatic carcinoma. By immunohistochemistry, breast markers were ER negative, PR negative and HER2neu equivocal and not amplified by fluorescence *in situ* hybridization (FISH). It is noteworthy that BRCA1 and BRCA2 were wild-type. Testing for germline mutations in 83 genes associated with genetic disorders was performed. Only one likely pathogenic variant was identified in the EGFR gene: c.2369 C>T {p. Thr790Met}. This mutation was heterozygous. Somatic alterations were checked using an assay that interrogates 324 genes and introns of 36 genes involved in rearrangements. Somatic alterations frequently observed in malignancies and variants of unknown significance were discovered (see **Table 1**). The tumor was Microsatellite stable, and Mutational Burden was 3 Muts/Mb. Tumor-infiltrating Immune Cell score was 10%, and Tumor Cell score was 0%. Tumor characteristics included that it was GATA-3 negative, CK7 patchy positivity with many cells showing a “dot-pattern” of staining, GCDFP-15 negative, Mammaglobin patchy moderately strong positivity in malignant cells with majority of cells negative, Synaptophysin positive, Chromogranin rare strong positivity in malignant infiltrate with “dot-pattern”, TTF-1 scattered positive cells, and CK20 negative. The patient declined testing of family members, primarily due to a history of Acute Lymphoblastic Leukemia in her child and fear of stigmatization.

**Table 1:** Results of somatic alterations and variants of unknown significance found in patient.

Gene	Alteration	Gene	Variant of Unknown Significance
EGFR	T790M	BRAF	E26D
PTEN	splice site 209 + 5G>A	IGF1R	N747S
RB1	loss	PTCH1	E48_N49insE
TP53	P58fs*65	SDHA	amplification
		CDKN2A/B	amplification
		IRS2	N28_H29insN
		RAF1	Q255P
		EPHA3	T406N
		NF1	S665F
		RB1	rearrangement
		FGF10	amplification
		PARK2	R191W
		RICTOR	amplification

Magnetic resonance imaging further characterized the previously biopsied right 10:00 breast lesion as irregular and lobulated with central areas of necrosis and heterogeneous enhancement predominantly in the periphery. The mass was measured 3 x 2.8 x 2.6 cm without skin invasion. Another mass was observed at 9:00 in the right breast, measuring 6 mm in greatest dimension and 2.7 cm away from the mass at 10:00. The previously biopsied right axillary LN was measured as 3.7 x 2.7 x 2.7 cm. There was also an 11 mm round LN with a near complete loss of normal fatty hilum superficial to the biopsy-proven metastatic LN in the axillary tail. At least six additional level I right axillary LNs were highly suspicious of metastatic LNs. Also, there was an abnormally enlarged level II LN, measuring 1.6 cm, that was suspicious for a metastatic LN. Three adjacent level II LNs were not enlarged, but did look suspicious relative to their left-sided counterparts. No internal mammary or level III lymphadenopathy was observed. A PET-CT showed abnormally increased FDG activity within the lateral aspect of the right breast and right axilla, but no other sites.

**Figure 1:**
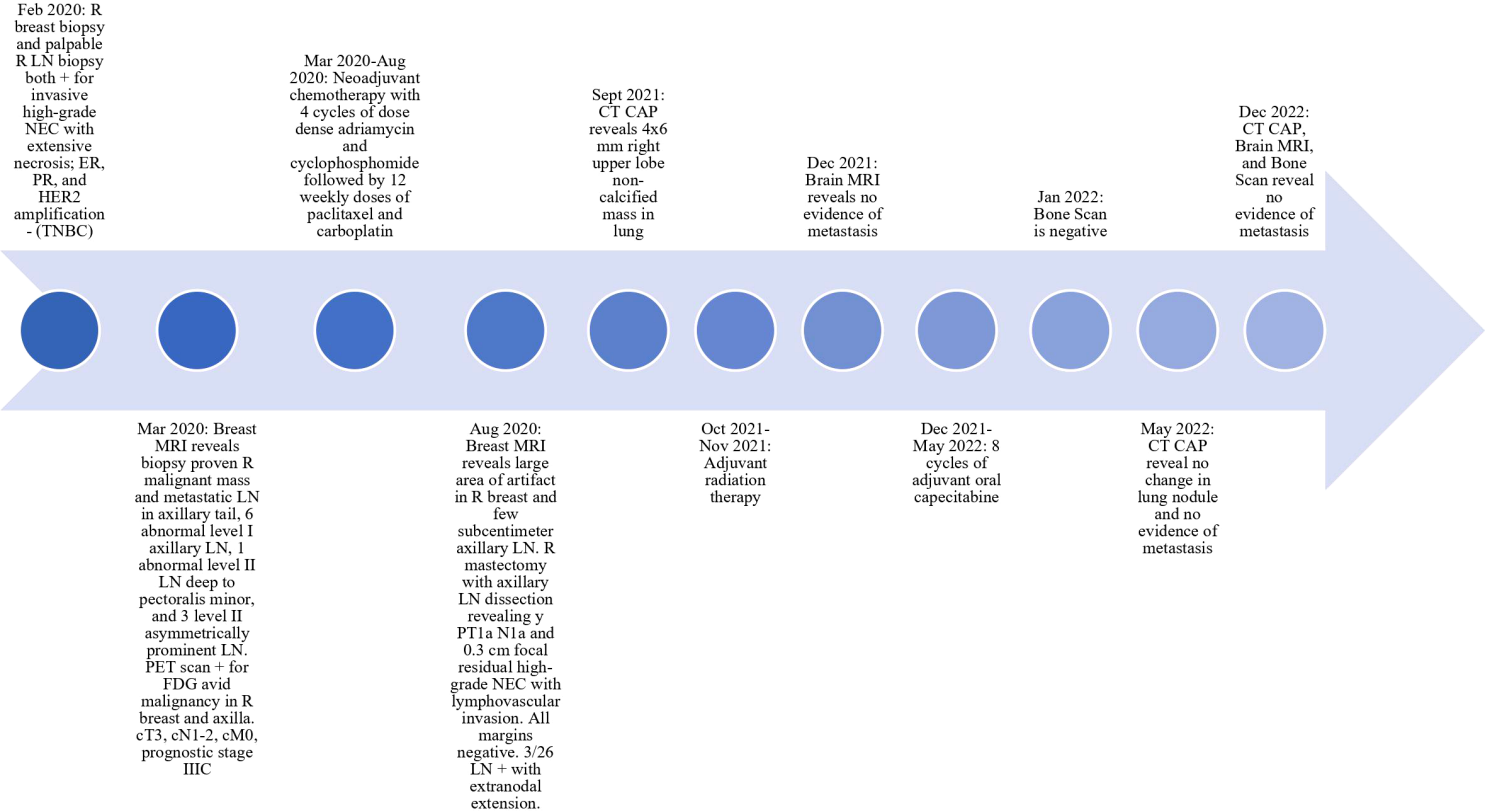
Timeline of Patient Diagnosis and Treatment. LN, lymph node; CT CAP, CT of chest/abdomen/pelvis; TNBC, triple negative breast cancer; NEC, neuroendocrine carcinoma.

The patient’s diagnosis was high-grade neuroendocrine carcinoma of the right breast (cT3 cN1-2 M0), clinical prognostic stage IIIC. Neoadjuvant chemotherapy consisting of four cycles of doxorubicin and cyclophosphamide followed by 12 cycles of paclitaxel and carboplatin was started. Carboplatin was discontinued after ten cycles due to neuropathy, which is managed with gabapentin. Once the chemotherapy regimen was completed, the patient’s breast mass was significantly decreased in size. The patient then underwent a right mastectomy with dissection of the right LNs, which showed residual ypT1aN1a. She developed lymphedema after surgery, but received treatment in the lymphedema clinic. A postoperative CTA-Chest showed multiple sub-centimeter indeterminate pulmonary nodules. She completed 25 adjuvant radiation fractions to the right chest wall and LN and developed grade 2 radiation dermatitis, which improved with treatment. The metastatic study completed after radiation showed no change in previously known sub-centimeter lung nodules. The patient then completed 8 cycles of adjuvant oral capecitabine. CT of chest/abdomen/pelvis done immediately after adjuvant chemotherapy revealed no change in lung nodules and no evidence of metastatic disease. CT of chest/abdomen/pelvis, brain MRI, and bone scan 7 months after adjuvant chemotherapy revealed no evidence of metastatic disease and stable appearance of chest (see **Figure 1**). The patient remains clinically disease free as of the last follow-up.

## Discussion

The EGFR gene is important in many different types of cancer. Most commonly found at exon 19, 20 or 21 of the EGFR gene, activating somatic mutations are important targets in the treatment of NSCLC with TKI and monoclonal antibody (3–14, 52). Additionally, the EGFR T790M mutation, located in exon 20 of the EGFR gene, was first discovered as a somatic resistance mechanism in patients with NSCLC who underwent treatment with first- or second-generation TKIs. A third-generation TKI, osimertinib, was approved in 2018 for patients with EGFR-mutated NSCLC, regardless of the status of the EGFR T790M mutation (22). Unfortunately, some patients also developed resistance to osimertinib through additional resistance mechanisms, including HER2 amplification and histologic transformation from NSCLC to high-grade, poorly differentiated, neuroendocrine lung cancer (18, 53–55).

Germline EGFR T790M mutations have been implicated in the development of lung cancer by acting as weak oncogenes (37, 38). Most patients with NSCLC and a germline EGFR T790M mutation also have a concurrent activating somatic EGFR mutation (35, 36). Only a minority of patients have a T790M mutation as their only EGFR mutation (33). Germline EGFR T790M mutations are estimated to occur in 1% of patients with NSCLC and between 0.15 and 0.54% of all cancer patients (29, 30, 38, 56). Germline mutations have been reported primarily in white patients, with few reported in those with East Indian or Asian ancestry (29, 36). This mutation is typically associated with a young age at the time of cancer diagnosis. It has been suggested that patients exhibit anticipation, with the youngest recorded patient diagnosed with this mutation and NSCLC at 29 years old (36). This highlights the importance of the EGFR T790M mutation in NSCLC as a resistance mechanism to treatment and also increases the risk of lung cancer without a significant predisposition to other types of cancer.

**Figure 2:**
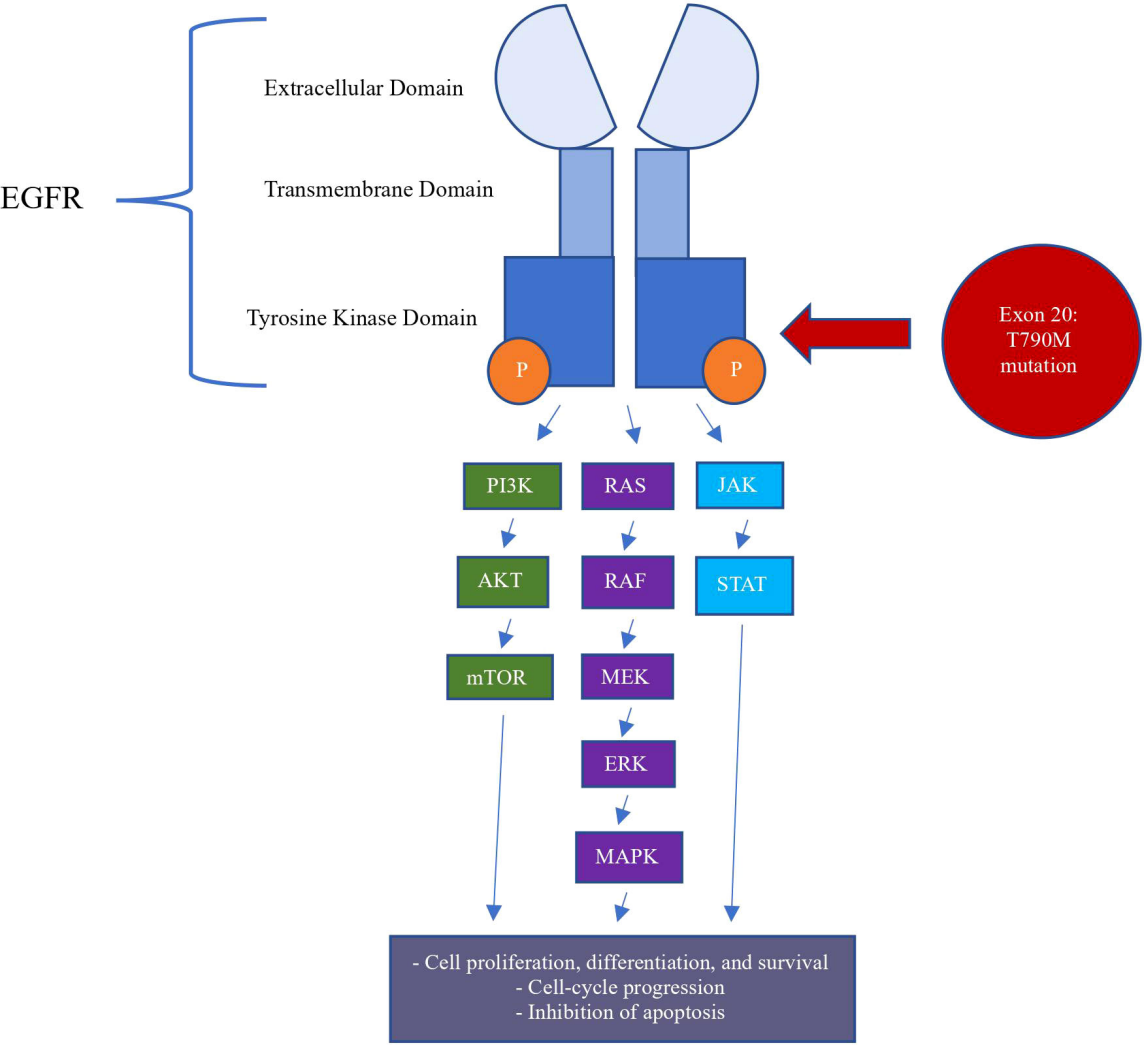
Intracellular Pathways of EGFR T790M Mutation in Pathogenesis of Breast Cancer.

Although the EGFR T790M mutation has been associated with NSCLC, there are only rare cases of the EGFR T790M mutation associated with BC. Teng et al. (50) reported EGFR mutations in 11.8% of 70 TNBC cases studied. The two most common mutations found were exon 19 deletions and exon 21 substitutions. In particular, no EGFR mutations were found at exon 20, which is the location of the EGFR T790M mutation (50) (see **Figure 2**). In a review of 131 BC patients, three patients were reported to have infiltrating ductal carcinoma with lone EGFR T790M mutations. While two of these patients had TNBC, all three mutations were somatic (51). Another interesting case is of a non-smoking woman with a history of BC who developed mutated lung adenocarcinoma; however, this mutation was not present in the breast connective tissue and was determined to be somatic (33).

The case we have presented is the first reported case of a germline EGFR T790M mutation in a patient with high-grade neuroendocrine TNBC. Since this is the first reported case, no association between the germline EGFR T790M mutation and TNBC can be made at this time. However, with the increasing amount of genetic testing performed on cancer patients and the ever-expanding testing options, there could be more cases in the future that would allow us to conclude germline EGFR T790M mutations and BC. Additional research on the EGFR T790M mutation may provide more treatment options for patients with any cancer mutated by EGFR T790M. This patient was not treated with a TKI, as no specific treatment guidelines are defined for breast neuroendocrine carcinomas (57). Still, third-generation TKIs may be a possible treatment option for them in the future based on their mutation. This case also highlights the importance of understanding the resistance mechanisms of the EGFR T790M mutation to third-generation TKI. Since one of the resistance mechanisms of EGFR T790M-mutated NSCLC is to histologically transform into high-grade, poorly differentiated, small cell lung cancer after treatment with osimertinib, studying this resistance mechanism may lead to a connection between the patient’s EGFR T790M mutation and neuroendocrine TNBC. Caution, however, will need to be exercised for such correlations as BC is a prevalent disease, and this is currently an incidental discovery.

There is a lack of high-quality data regarding therapeutic options in patients with disease progression of neuroendocrine carcinoma of the breast (58). Neuroendocrine carcinoma follows a more aggressive course than ductal carcinoma (57, 59). Options for treatment include chemotherapy according to neuroendocrine malignancy guidelines (60). There are case reports of response with immunotherapy (61, 62); however, there is no well-described correlation with conventional predictors of response to immunotherapy such as PD-L1 expression, high tumor mutational burden, and microsatellite instability. There are reports of response with peptide receptor radionuclide therapy in patients with metastatic neuroendocrine breast carcinoma and overexpression of somatostatin receptors as evidenced by nuclear scintigraphy (63, 64). The somatic PTEN mutation in this patient also provides the theoretical option of using mTOR inhibition for clinical benefit (65). TROP-2 protein expression has been observed in some patients with neuroendocrine breast carcinoma, suggesting a role for targeted therapy with sacituzumab govitecan (66, 67). Similarly, predictive expression of FOLR1 and H3K36Me3 has been observed in subsets of neuroendocrine BC, which may pave the way for future usage of newer drugs such as farletuzumab and mirvetuximab soravtansine (FOLR1) and histone deacetylase inhibitors (H3K36Me3) (66).

This case also highlights the complicated nature of genetic testing. While specific mutations have provided patients with life-changing treatments, there are many other times when a mutation is found with no particular significance at the time of the test or when the mutations found lead to more questions than answers. In the case of our patient, these new questions revolve around what other cancer screening must be done beyond screening and surveillance of her known disease. There are no standard guidelines for cancer screening in patients with identified EGFR T790M mutations. Patients who have germline EGFR T790M mutations require further studies to assess the presence of lung cancer. Our patient is no different due to her germline mutation and the multiple sub-centimeter indeterminate pulmonary nodules found on her postoperative CTA chest. Although these could be benign, serial imaging should be done to properly characterize these nodules. However, this patient does not have any other EGFR mutations, which is a rarity in germline EGFR T790M-mutated NSCLC patients (33). While no significance can be tied to this currently, this can affect her risk of developing lung cancer. A detailed family history is essential, as this germline mutation is most commonly associated with lung adenocarcinoma. The unknown paternal health history of this patient could provide more information about her case. Furthermore, patients with germline EGFR T790M mutations should seek genetic counseling for themselves and their first-degree relatives. Oxnard et al. (35) also recommend following those with germline EGFR T790M to determine optimal screening and counseling strategies. As more is learned about the EGFR T790M mutation, screening protocols for patients with germline EGFR T790M mutations should be clearly defined to increase the chance of survival of these patients and possibly their families.

## Conclusion

This case is unique due to the patient’s presentation and her mutation status. At this time, no association can be made between BC and EGFR T790M mutation. Further investigation of the EGFR T790M mutation could elucidate an increased risk of breast or other types of cancer beyond NSCLC. Additional uses for TKIs could also be found along with defined cancer screening protocols to benefit patients who have a germline EGFR T790M mutation. Continued research on the EGFR T790M mutation will help this patient and many others today and in the future with their cancer prevention and treatment.

## Patient perspective

The patient had struggled emotionally throughout the process. She had developed grade 1 neuropathy while receiving adjuvant chemotherapy and palmar-plantar erythrodysesthesia while receiving capecitabine.

## Data availability statement

The original contributions presented in the study are included in the article/supplementary material. Further inquiries can be directed to the corresponding author.

## Ethics statement

The authors have obtained written informed consent from the patient for the publication of this case history and applicable data.

## Author contributions

OS contributed to the review of the literature and the writing and editing of the case report. AR contributed to the review of the literature and the writing and editing of the case report. BJ contributed to the editing of the case report. MH contributed to reviewing the case history. AA contributed to reviewing the case history. MR was critical to the direction, editing, and submission of the case report. All authors contributed to the article and approved the submitted version.
